# 18S rDNA sequence-structure phylogeny of the eukaryotes simultaneously inferred from sequences and their individual secondary structures

**DOI:** 10.1186/s13104-024-06786-9

**Published:** 2024-05-01

**Authors:** Eva Rapp, Matthias Wolf

**Affiliations:** Department of Bioinformatics, Biocenter, University of Würzburg, Würzburg, Germany

**Keywords:** Eukaryotes, Phylogenetics, 18S rDNA, Secondary structure

## Abstract

**Objective:**

The eukaryotic tree of life has been subject of numerous studies ever since the nineteenth century, with more supergroups and their sister relations being decoded in the last years. In this study, we reconstructed the phylogeny of eukaryotes using complete 18S rDNA sequences and their individual secondary structures simultaneously. After the sequence-structure data was encoded, it was automatically aligned and analyzed using sequence-only as well as sequence-structure approaches. We present overall neighbor-joining trees of 211 eukaryotes as well as the respective profile neighbor-joining trees, which helped to resolve the basal branching pattern. A manually chosen subset was further inspected using neighbor-joining, maximum parsimony, and maximum likelihood analyses. Additionally, the 75 and 100 percent consensus structures of the subset were predicted.

**Results:**

All sequence-structure approaches show improvements compared to the respective sequence-only approaches: the average bootstrap support per node of the sequence-structure profile neighbor-joining analyses with 90.3, was higher than the average bootstrap support of the sequence-only profile neighbor-joining analysis with 73.9. Also, the subset analyses using sequence-structure data were better supported. Furthermore, more subgroups of the supergroups were recovered as monophyletic and sister group relations were much more comparable to results as obtained by multi-marker analyses.

**Supplementary Information:**

The online version contains supplementary material available at 10.1186/s13104-024-06786-9.

## Introduction

The eukaryotic tree of life was and still is object to changes: from the former classification of the eukaryotes into “kingdoms” cf. [1] to the current supergroups most recently reviewed by Keeling and Burki [2] and Burki et al. [3]. One of the most frequently sequenced genes in eukaryotes is the 18S ribosomal deoxyribonucleic acid (18S rDNA) [4]. However, due to its length-variable regions, alignments, in particular on a large taxonomic scale, show ambiguities and are leading to inconsistencies regarding any phylogenetic reconstruction [4]. Further, 18S rDNA sequences often are not complete and only partially available on NCBI [5]. This makes a well-balanced taxon sampling over all eukaryotes difficult, especially when you only want to use full-length sequences simultaneously with information as obtained from their individual secondary structures. According to Keller et al. [6] the simultaneous usage of RNA sequences and their individual secondary structure increases robustness and accuracy of phylogenetic analyses. Sequence-structure data (encoded in a new alphabet) have already been used in several case studies [7–16]. In this study we only use complete 18S ribosomal ribonucleic acid (rRNA) gene sequences and their individual secondary structures, as obtained from RNAcentral [17], and additionally curated manually by the Comparative RNA Web Site (CRW) [18]. For an automatic approach this is still the best data set available, despite that the taxon sampling is not perfectly balanced and several higher taxa are missing.

## Main text

### Methods

#### Taxon sampling

In the supplementary information we provide a flowchart of the used methods and the resulting figures. Cytosolic 18S rDNA sequences and their individual secondary structures, curated by the Comparative RNA Web [18], were obtained from RNAcentral [17] (retrieved on 06/06/2023).

In total, sequence-structure data for 215 taxa were acquired. Four taxa were removed from the dataset; two showed uneven length concerning the primary sequence and the respective secondary structure information (the latter being provided in dot bracket notation) and two were classified as possibly contaminated. A subset of 47 taxa was manually chosen, representing the overall dataset proportionally. A list with species names and GenBank accession numbers of all taxa can be found in the Additional file 1.

#### Alignments

**Fig. 1:**
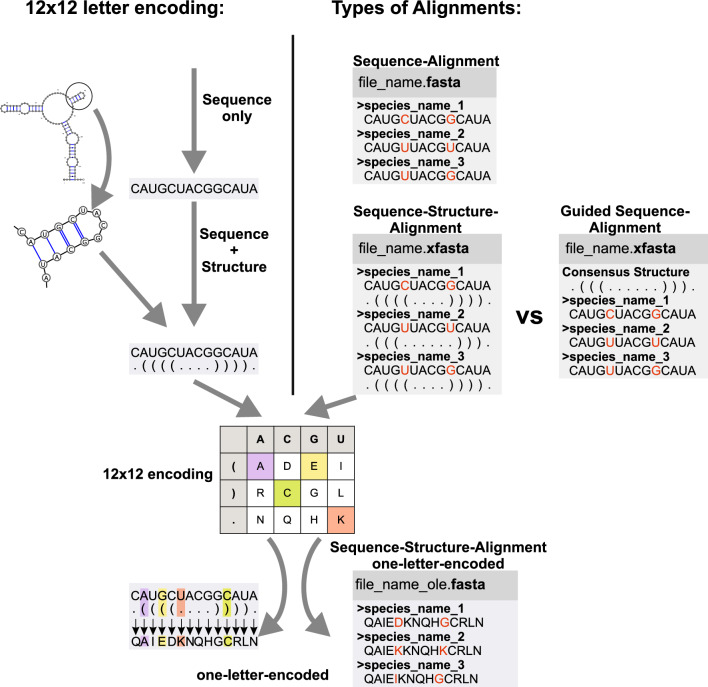
Left: Encoding of sequence-structure information. Scoring matrices and substitution models have been adapted accordingly. The figure shows an RNA sequence with its individual secondary structure in the bracket-dot-bracket notation. The respective 2D structure, the 12-letter translation table as well as the one-letter-encoded pseudoprotein sequence are depicted. Right: Different alignments are shown. They differ in terms of informational content (exemplarily highlighted in red). Only the sequence-structure-alignments as derived from 4SALE [20–22] include information about individual secondary structures whereas the guided-sequence alignment is guided only by a consensus structure

For the two datasets four alignments were constructed. Either sequence-only alignments using ClustalX [19] or sequence-structure alignments using ClustalW [19] as implemented in 4SALE [20, 21]. 4SALE [20–22] uses a 12-letter translation table to encode the sequence-structure information into a one-letter-encoded pseudoprotein sequence. (cf. Figure 1). Pseudoprotein sequences are automatically aligned using a 12 × 12 scoring matrix [20–22].

#### Tree reconstruction

**Fig. 2:**
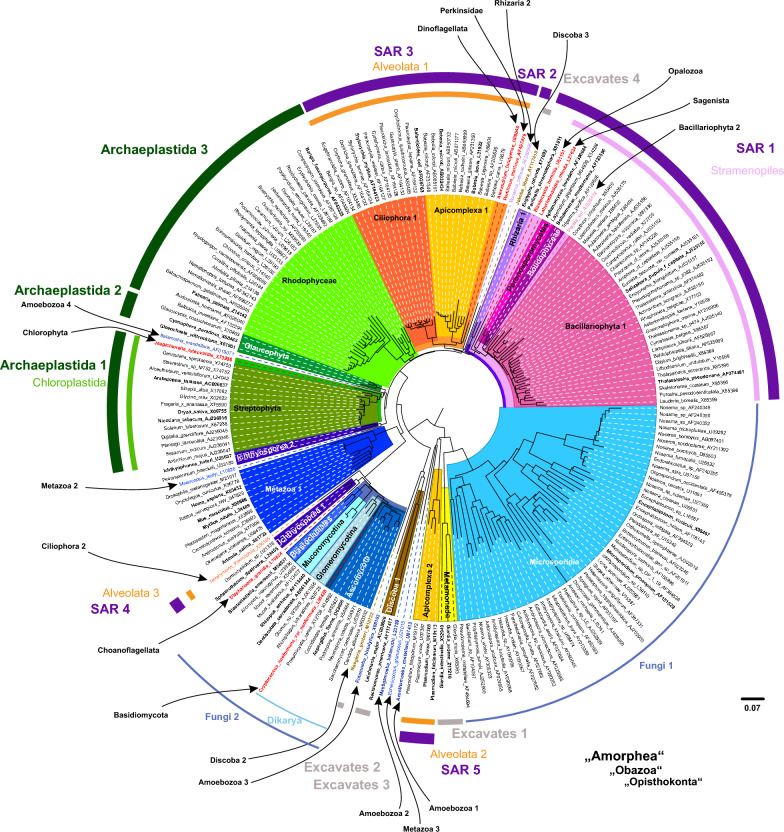
Overall sequence-structure NJ tree using the 18S rDNA of all 211 taxa. ClustalW [19], as implemented in 4SALE [20, 21], was used for the global multiple sequence-structure alignment. The tree was reconstructed using ProfDistS [25, 26] and midpoint rooted. The scale bar shows evolutionary distances. Taxa names are accompanied by their corresponding GenBank accession number. Clades and respective singular taxa are marked in a color-scheme based on the eukaryotic tree of life published by Keeling and Burki [2]. If clades and singular taxa do not form one monophyletic group, they are numbered consecutively. If a group is only represented by one taxon, the taxon is marked in red. Taxa which were manually selected for the subsampling are marked bold. Supergroups are indicated according to Burki et al. [3] and Keeling and Burki [2], the names of the supergroups are adapted based on Adl. et al. [27]. With regards to readability the supergroups Amorphea, Obazoa and Opisthokonta are only named once near the biggest monophyletic subgroup. The three supergroups are marked with quotation marks since they are not monophyletic. The supergroup Opisthokonta includes Fungi, Metazoa, Choanoflagellata and Ichthyosporea. Amoebozoa are classified as Obazoa

**Fig. 3:**
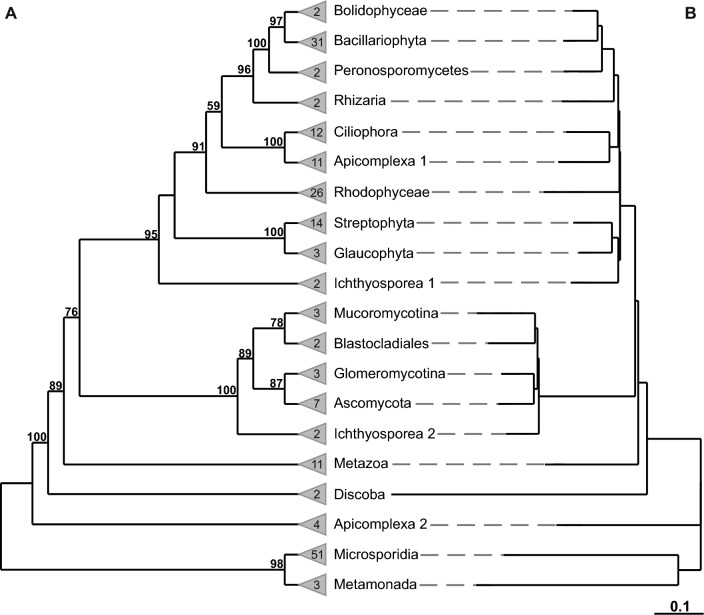
Two-times iterated sequence-structure PNJ tree with BS values (**A**) and original BL (**B**). Profiles were predefined according to Fig. 2; singletons were not included. The scale bar shows evolutionary distances. The trees were reconstructed using ProfDistS [25, 26] and rooted according to the overall sequence-structure NJ tree (Fig. 2). In each iteration, super-profiles of profiles have been built based on BS values (> 75). At internodes, the BS values from 100 pseudo-replicates have been mapped. The numbers in the triangles in front of the taxa represent the quantity of taxa included in the profile

The overall sequence-only neighbor-joining [23] (NJ) tree (Additional file 1) and the overall sequence-structure NJ tree (Fig. 2) as well as the corresponding profile neighbor-joining [24] (PNJ) trees (Additional file 1 and Fig. 3) were reconstructed using ProfDistS [25, 26]. Supergroups were indicated in the trees according to Burki et al. [3] and Keeling and Burki [2], the names of the supergroups are adapted based on Adl. et al. [27].

According to Müller et al. [24], Friedrich et al. [25], Rahmann et al. [28] and Wolf et al. [26], the basal branching patterns of very large trees often cannot be estimated unambiguously. The PNJ algorithm, which is implemented in ProfDistS [25, 26], estimates the tree topology for defined profiles of subclades, independent of the topology within each subclade [24–26, 28]. Profiles for each PNJ estimation were predefined according to the overall NJ tree (Additional file 1 and Fig. 2). PNJ trees (Additional file 1 and Fig. 3) were reconstructed in two iterations. Bootstrap (BS) support [29] was estimated, due to the complexity of the sequence-structure approach, using only 100 pseudo-replicates.

The manually chosen subset of the 47 taxa was further processed using sequence-only as well as sequence-structure NJ-, maximum parsimony [30] (MP) and maximum likelihood [31] (ML) analyses. BS support for all the subset trees was estimated using 100 pseudo-replicates. The sequence-only NJ (Additional file 1) as well as the sequence-structure NJ (Additional file 1) trees were reconstructed using ProfDistS. The sequence-only MP (Additional file 1) and the sequence-structure MP (Additional file 1) tree as well as the sequence-only ML trees with BS (Additional file 1) and branch lengths (BL) (Additional file 1) were reconstructed with PAUP* 4.0a [32] using default settings. Using phangorn [33] as implemented in R [34], the sequence-structure ML trees with BS and BL were reconstructed using a GTR + I + G substitution model. The R script is available at the 4SALE homepage [20].

#### Prediction of consensus structures

Based on the sequence-structure alignment of the subset, the 75% and 100% consensus structures were predicted using a python script. The python script is available on the 4SALE homepage (https://4sale.bioapps.biozentrum.uni-wuerzburg.de). Using Pseudoviewer [35], the 75% consensus structure was drawn and the 100% consensus structure was then marked within the resulting 75% consensus figure (Additional file 1). In addition, both consensus structures were mapped on the structure of *Homo sapiens* (Additional file 1), available on RNAcentral [17].

## Results

### Overall neighbor-joining trees

#### Sequence-only

An overall sequence-only NJ tree (Additional file 1) based on 211 sequences was reconstructed with ProfDistS [25, 26] and rooted at its midpoint.

With regards to the supergroups according to Keeling and Burki [2] and Burki et al. [3], only Stramenopiles, Rhizaria and Metamonada were recovered as monophyletic. The other supergroups were non-monophyletic: The SAR group as well as Archaeplastida split in three clades each. Amorphea, consisting of nine Opisthokonta clades and four single Opisthokonta taxa as well as one Amoebozoa clade and two Amoebozoa singletons, separated into 10 clades and six singletons in total. Excavates split into two clades and two singletons.

Several groups within the non-monophyletic supergroups were recovered as monophyletic including Ciliophora, Rhodophyceae, Chloroplastida and Glaucophyta as well as Mucoromycotina, Dikarya, Glomeromycotina and Blastocladiales. Glomeromycotina and Dikarya are sister groups.

#### Sequence-structure

Additionally, to the sequence-only NJ tree (Additional file 1), an overall sequence-structure NJ tree (Fig. 2) was reconstructed with ProfDistS and midpoint rooted.

Out of the supergroups according to Keeling and Burki [2] and Burki et al. [3], only Stramenopiles and Metamonada were recovered as monophyletic. The other supergroups were non-monophyletic: The SAR group separated into 5 groups. Corresponding to the sequence-only NJ tree, Archaeplastida split into three clades. Amorphea separated into five clades and seven singletons: four single Amoebozoa taxa and five Opisthokonta clades as well as three Opisthokonta singletons. Excavates split into four clades.

The groups which were recovered as monophyletic within the non-monophyletic supergroups are: Rhodophyceae, Glaucophyta, Chloroplastida and Microsporidia as well as a monophyletic clade within Amorphea. This clade consisted of the each monophyletic Dikarya plus Blastocladiales, Mucoromycotina and Glomeromycotina.

The sister group relations of the overall NJ trees are described in the following together with the results of the PNJ analyses.

### Profile neighbor-joining trees

Fifteen taxa from the sequence-only NJ tree (Additional file 1) and eighteen taxa from the sequence-structure NJ tree (Fig. 2) were excluded from predefined profiles for the PNJ analyses, since they could not be unambiguously assigned to a subclade in the respective overall NJ tree. Based on the subclades from the respective NJ trees 23 profiles for the sequence-only PNJ analysis and 20 profiles for the sequence-structure PNJ analysis were defined.

#### Sequence-only PNJ tree

The sequence-only PNJ tree (Additional file 1) showed generally lower bootstrap support at the basal branches and the SAR group as well as the Archaeplastida and Opisthokonta did not form the same clades as in the sequence-structure PNJ tree (Fig. 3) (cf. discussion).

#### Sequence-structure PNJ tree

Except for Apicomplexa 2 (*Plasmodium* clade), which was located at the base of the tree and was represented by four taxa, all other members of the SAR group were recovered as a monophylum with low support (59 = bootstrap support) in the two-times iterated PNJ tree (Fig. 3A). Stramenopiles, consisting of two bolidophycean and 31 taxa of Bacillariophyta and two Peronosporomycetes, were fully supported (100). Stramenopiles formed a well-supported (96) sister clade to Rhizaria, which was represented by two taxa. Alveolata, consisting of 12 Ciliophora taxa and 11 Apicomplexa 1 (*Babesia* clade) taxa, was positioned at the base of the Stramenopiles clade and was fully supported (100).

Out of the Archaeplastida, only Streptophyta, represented by 14 taxa, and Glaucophyta, represented by three taxa, formed a fully supported (100) clade. Rhodophyceae was represented by 26 taxa and formed a well-supported (91) sister clade to the SAR clade.

A fully supported (100) “big Opisthokonta clade” is sister to the SAR clade plus Archaeplastida, plus Rhodophyceae and Ichthyosporea 2 (*Ichthyophonus* plus *Psorospermium*). Ichthyosporea 2 forms a well-supported (95) sister clade to the Archaeplastida clade plus Rhodophyceae and the SAR clade. The Opisthokonta clade consists of Ichthyosporea 1 (*Dermocystidium* plus *Sphaerothecum*) and its well-supported (89) sister, the monophyletic Fungi clade, formed by Mucoromycotina, Blastocladiales, Glomeromycotina and Ascomycota. Metazoa is represented by 11 taxa and is the well-supported (89) sister clade to SAR/Archaeplastida/Rhodophyceae/Opisthokonta/Ichthyosporea2.

The Excavates do not form a monophylum. The PNJ tree was rooted according to the respective NJ tree at its midpoint and therefore Microsporidia plus Metamonada formed a sister group to the remaining taxa. Discoba is represented by two taxa and is the fully supported (100) sister to SAR/Archaeplastida/Rhodophyceae/Opisthokonta/Ichthyosporea2/Metazoa.

The original sequence-structure PNJ tree (Fig. 3B) as well as the iterated PNJ tree (Fig. 3A) showed the same topology.

The position of Ichthyosporea 2 varied between the original NJ tree and the respective PNJ tree: While it was a sister clade to the SAR clade plus Archaeplastida and Rhodophyceae in the PNJ tree, it forms a sister clade to Chloroplastida plus the amoebozoan *Balamuthia* and Glaucophyta in the NJ tree.

The average BS per node for the sequence-structure PNJ tree with around 90.3 was higher than the average BS per node for the sequence-only PNJ tree with 73.9.

### Subsampling (ML/MP/NJ)

47 taxa from the overall NJ trees (Additional file 1 and Fig. 2) were manually chosen as a subset and newly aligned. The alignments were further processed using ML, MP and NJ analyses and the respective trees were reconstructed and rooted according to the overall NJ trees. BS support was estimated using 100 pseudo replicates. Subsample trees (sequence-only and sequence-structure) are available as supplementary information and thoroughly described therein, together with the consensus structures of the subsample sequence-structure alignment.

## Discussion

### Overall NJ trees

Regarding the recent studies by Keeling and Burki [2] as well as Burki et al. [3] concerning the phylogeny of the eukaryotes, the supergroup Rhizaria, which is monophyletic in the overall sequence-only NJ tree (Additional file 1), splits into a single taxon and one clade in the sequence-structure NJ tree (Fig. 2). The monophyletic Ciliophora split in a singleton and one clade in the sequence-structure approach. One improvement in the sequence-structure NJ tree, compared to the sequence-only NJ tree, is that Microsporidia were recovered as monophyletic. Additionally, a big monophyletic Fungi clade within Opisthokonta was recovered in the sequence-structure tree.

The differences regarding sister group relations of the overall NJ trees are discussed in the following together with the results of the PNJ analyses.

### PNJ trees

The backbone of both PNJ trees (Additional file 1 and Fig. 3), whose profiles were defined according to the overall NJ tress (Additional file 1 and Fig. 2), shows differences: the overall profiles of both PNJ trees vary regarding their positions to each other.

With the singletons of the NJ trees being left out in the PNJ analyses, this study shows, that the sequence-structure PNJ tree with an average BS of 90.3, is generally better supported than the sequence-only PNJ tree, which had an average BS of 73.9. Additionally to showing higher support, several of the supergroups according to Keeling and Burki [2] and Burki et al. [3] were recovered in bigger monophyletic clades in the sequence-structure PNJ tree compared to the sequence-only PNJ approach: the SAR group, Opisthokonta and Archaeplastida. Furthermore, the SAR clade is sister to both Archaeplastida clades. The three Opisthokonta clades are sister to the SAR clade plus Archaeplastida. The same sister group relations are also shown in the study by Burki et al. [3].

### ML trees with BS from ML, MP and NJ analyses

While both ML trees, the sequence-only as well as the sequence-structure approach, recovered the same three supergroups as monophyletic (Metamonada, Stramenopiles and Rhizaria), the sequence-structure ML tree shows several differences, which are closer to the results of the studies by Keeling and Burki [2] and Burki et al. [3], and also higher BS support: With BS values of 56 (MP) and 54 (ML), the backbone of the sequence-only MP (Additional file 1) and the sequence-only ML (Additional file 1) tree showed nearly no support.

The Opisthokonta, which split into four clades and three singletons in the sequence-only ML tree, were reconstructed as one big monophyletic clade and two singletons in the sequence-structure ML tree. This big Opisthokonta clade of the sequence-structure approach also showed moderate MP (69) and high NJ (98) BS support.

The Archaeplastida split into the same three clades in the sequence-only ML tree as well as in the sequence-structure tree: Glaucophyta, Chloroplastida and Rhodophyceae. The BS support for each of the three clades was higher in the sequence-structure approaches: only the MP BS support for the Glaucophyta clade as well as the Chloroplastida clade was lower than 100, with a BS support of 98. Additionally, the members of Archaeplastida showed closer sister group relations to the members of Opisthokonta in the sequence-structure approach.

While the members of the SAR group did not even form sister groups in the sequence-only ML tree, the SAR group was nearly monophyletic in the sequence-structure ML tree, except for one Apicomplexa clade. Stramenopiles and Rhizaria were monophyletic in both approaches, with Rhizaria being fully supported. Stramenopiles, nevertheless, showed only moderate support in the sequence-only approaches but was fully supported in the sequence-structure trees. Alveolata split into five clades in the sequence-only ML tree and was recovered as a big monophylum, except for the before mentioned Apicomplexa clade, in the sequence-structure ML tree. This big Alveolata clade was additionally highly supported (95/95/99) (= bootstrap support from ML/MP/NJ analyses).

The Excavates were recovered at the base of the trees and as non-monophyletic in the sequence-only as well as in the sequence-structure approaches.

With more/bigger monophyletic supergroups or monophyletic clades within the supergroups, as well as regarding the sister group relations, the sequence-structure approaches show more resemblance to the eucaryotic trees of life by Keeling and Burki [2] and Burki et al. [3]. Phylogenetic analyses using RNA or protein data generally benefit from the inclusion of structural data [6, 38].

### Consensus structures

The 75 and 100 percent consensus structures of the subset (Additional file 1), which were predicted in this study, show, that almost all helices (variable regions are named according to Dams et al. [36]) contain 75 percent conserved nucleotide pairs, with V5 and V7-V9 being the most conserved variable regions. V1 and V3 contain the 100 percent conserved nucleotide pairs. Regarding the location of conserved nucleotide pairs and the universally conserved bases of the eukaryotes according to Noller et al. [37], regions with universally conserved bases coincide with conserved nucleotide pairs (Additional file 1). This suggests good quality of the data and of the alignment, which were used in this study.

### Limitations

- The root for the eukaryotic tree of life is under debate and a midpoint root is merely a stopgap solution.
- A perfectly balanced taxon sampling for a simultaneous sequence-structure analysis is unfortunately not possible due to the current data situation.

### Supplementary Information

**Additional file 1. **Flowchart of the workflow, supplementary trees, consensus structures, and GenBank accession numbers.

## Data Availability

The datasets used and/or analyzed during the current study are available from the corresponding author on reasonable request.

## Abbreviations

BL: Branch lengths
BS: Bootstrap
CRW: Comparative RNA Web
ML: Maximum likelihood
MP: Maximum parsimony
NJ: Neighbor-joining
PNJ: Profile neighbor-joining
rDNA: Ribosomal deoxyribonucleic acid
rRNA gene: Ribosomal ribonucleic acid gene
