# Supplementary material for: 18S rDNA sequence-structure phylogeny of the eukaryotes simultaneously inferred from sequences and their individual secondary structures
Source: BMC Res Notes. 2024 May 1;17:124. doi: 10.1186/s13104-024-06786-9 (PMC11064340; doi:10.1186/s13104-024-06786-9)
Supplement: Supplementary file 1 — Additional file 1. Flowchart of the workflow, supplementary trees, consensus structures, and GenBank accession numbers. [file 13104_2024_6786_MOESM1_ESM.pdf]

Additional file 1

Supplementary material

18S rDNA sequence-structure phylogeny of the eukaryotes simultaneously inferred from sequences and their individual secondary structures available on the Comparative RNA Web Site

Eva Rapp (eva.rapp@uni-wuerzburg.de)

Department of Bioinformatics, Biocenter, University of Würzburg, Würzburg, Germany

Matthias Wolf (matthias.wolf@uni-wuerzburg.de)

Department of Bioinformatics, Biocenter, University of Würzburg, Würzburg, Germany

Supplementary Results

Subsample sequence-only tree

In the sequence-only ML tree with BL (Fig. S9) only Metamonada, Stramenopiles and Rhizaria out of the supergroups according to Keeling and Burki [1] and Burki et al. [2], were recovered as monophyletic. The backbone of the MP (Fig. S7) and the ML (Fig. S8) tree showed almost no support with BS values of 56 (MP) and 54 (ML).

The Opisthokonta split into four clades and three singletons. Microsporidia were positioned at the base of the tree. Within the Opisthokonta, the Metazoa formed a monophyletic clade and showed high support (78/100/100) in all three trees (= bootstrap support from ML/MP/NJ analyses). Ichthyosporea plus Choanoflagellata formed another monophyletic Opisthokonta clade as well as Mucoromycotina plus Blastocladiales. Those two Opisthokonta clades together with Glomeromycotina plus Basidiomycota and Ascomycota as well as one taxon classified within Amoebozoa formed a big Obazoa clade. Amoebozoa were non-monophyletic.

Archaeplastida were non-monophyletic and split into three clades. One consisted of the highly supported (97/82/100) monophyletic Rhodophyceae, the second of the fully supported (100/99/100) monophyletic Chloroplastida and the third of the monophyletic Glaucophyta, which were highly supported (89/100/96).

The SAR group was non-monophyletic. Within the SAR group only the moderately supported (88/63/-) Stramenopiles and the fully supported (100/100/100) Rhizaria were monophyletic, but they did not form sister groups. The Alveolata were non-monophyletic and split into 5 clades.

Within the non-monophyletic Excavates, the fully supported (100/100/100) monophyletic Metamonada formed a not supported sister clade to a single taxon classified within Discoba. Discoba was not monophyletic and split into three clades.

Subsample sequence-structure tree

Out of the supergroups according to Keeling and Burki [1] and Burki et al. [2], Metamonada, Stramenopiles and Rhizaria were recovered as monophyletic.

All members of the Opisthokonta except for Microsporidia and Ichthyosporea 2 (*Ichthyophonus)* grouped in one big clade with no ML BS support but moderate MP and high NJ BS support (36/69/98). Within the Opisthokonta the Metazoa formed a fully supported sister clade (100/100/100) to another fully supported (100/100/100) clade consisting of Mucoromycotina, Ascomycota, Glomeromycotina, Basidiomycota, Ichthyosporea 1 (*Sphaerothecum*), Choanoflagellata and Blastocladiales. Ichthyosporea 2 grouped together with Glaucophyta with low ML and MP support and high NJ support (52/59/92) and formed a not supported sister clade to "the big Opisthokonta clade". The non-monophyletic Amoebozoa formed three clades near the base of the tree.

The Archaeplastida are non-monophyletic and split into three clades. The Glaucophyta formed a monophyletic clade with high support (100/98/100). The monophyletic Streptophyta were fully supported (100/100/100) as well and together with its sister Chlorophyta they formed the monophyletic Chloroplastida with high support (100/98/100). The Chloroplastida were sister to "the big Opisthokonta clade" plus Glaucophyta plus Ichthyosporea 2 with no support. Rhodophyceae were also monophyletic with full support (100/100/100). They were sister to the clade consisting of "the big Opisthokonta clade" plus Glaucophyta and Ichthyosporea 2 but with no support.

Nearly all members of the SAR group except for Apicomplexa 2 (*Plasmodium* clade), grouped as a not supported monophylum and formed the not supported sister to “the big Opisthokonta clade” plus Glaucophyta, Ichthyosporea 2, Chloroplastida and Rhodophyceae. Within the SAR group, the Stramenopiles grouped as a monophylum with full support (100/100/100) and were the not supported sister to a big, highly supported (95/95/99) Alveolata clade consisting of Perkinsidae, Dinoflagellata, Apicomplexa 1 (*Babesia* clade) and Ciliophora. The monophyletic Apicomplexa 1 and the monophyletic Ciliophora clade were each fully supported (100/100/100). The fully supported (100/100/100) Rhizaria were sister to Alveolata plus Stramenopiles but with no support.

The Excavates were not monophyletic. The monophyletic and fully supported (100/100/100) Metamonada were positioned at the base of the tree. Discoba (non-monophyletic) formed three clades also near the base of the tree next to Ameobozoa 3 (*Entamoeba*).

Consensus structures of subsample

The 75 and 100 percent consensus structures were predicted for the 47 subset taxa (Fig. S5). The consensus structure is 1751 bases long. 293 nucleotide pairs are at least 75 percent conserved. 22 out of the 293 pairs are 100 percent conserved. Therefore 271 pairs are only 75 percent conserved.

The 100 percent conserved nucleotide pairs are located in the variable regions V1 and V3 (variable regions are named according to Dams et al. [3]). All helices show at least some 75 percent conserved nucleotide pairs. Especially the structure in the variable regions V5 and V7-V9 are the most conserved ones.

Figure S10 of the supplement shows the predicted 75 percent consensus structure, which was mapped on the secondary structure of *Homo sapiens*. The universally conserved bases of the eukaryotes according to Noller et al. [4] have been marked in the figure. All helices contain

universally conserved bases. Especially regions V1, V3, V5 and V7-V9 showed several of the universally conserved bases.

References

1. Keeling PJ, Burki F. Progress towards the Tree of Eukaryotes. Current Biology. 2019. doi:10.1016/j.cub.2019.07.031.
2. Burki F, Roger AJ, Brown MW, Simpson AGB. The New Tree of Eukaryotes. Trends in Ecology & Evolution. 2020. doi:10.1016/j.tree.2019.08.008.
3. Dams E, Hendriks L, van de Peer Y, Neefs JM, Smits G, Vandenbempt I, Wachter R de. Compilation of small ribosomal subunit RNA sequences. Nucleic Acids Research. 1990. doi:10.1093/nar/18.suppl.2237.
4. Noller HF, Donohue JP, Gutell RR. The universally conserved nucleotides of the small subunit ribosomal RNAs. RNA. 2022. doi:10.1261/rna.079019.121.
5. Cannone JJ, Subramanian S, Schnare MN, Collett JR, D'Souza LM, Du Y, et al. The Comparative RNA Web (CRW) Site: An Online Database of Comparative Sequence and Structure Information for Ribosomal, Intron, and Other RNAs. BMC Bioinformatics 2002. doi:10.1186/1471-2105-3-2.
6. RNAcentral Consortium. RNAcentral: an international database of ncRNA sequences. Nucleic Acids Research. 2015. doi:10.1093/nar/gku991.
7. Larkin MA, Blackshields G, Brown NP, Chenna R, McGettigan PA, McWilliam H, et al. ClustalW and ClustalX Version 2.0. Bioinformatics. 2007. doi:10.1093/bioinformatics/btm404.

8. Seibel PN, Müller T, Dandekar T, Schultz J, Wolf M. 4SALE – a tool for synchronous RNA sequence and secondary structure alignment and editing. BMC Bioinformatics. 2006. doi:10.1186/1471-2105-7-498.
9. Seibel PN, Müller T, Dandekar T, Wolf M. Synchronous visual analysis and editing of RNA sequence and secondary structure alignments using 4SALE. BMC Research Notes. 2008. doi:10.1186/1756-0500-1-91.
10. Saitou N, Nei M. The neighbor-joining method: a new method for reconstructing phylogenetic trees. Molecular Biology and Evolution. 1987. doi:10.1093/oxfordjournals.molbev.a040454.
11. Müller T, Rahmann S, Dandekar T, Wolf M. Accurate and robust phylogeny estimation based on profile distances: a study of the Chlorophyceae (Chlorophyta). BMC Evolutionary Biology. 2004. doi:10.1186/1471-2148-4-20.
12. Friedrich J, Dandekar T, Wolf M, Müller T. ProfDist: a tool for the construction of large phylogenetic trees based on profile distances. Bioinformatics. 2005. doi:10.1093/bioinformatics/bti289.
13. Wolf M, Ruderisch B, Dandekar T, Schultz J, Müller T. ProfDistS: (profile-) distance based phylogeny on sequence-structure alignments. Bioinformatics. 2008. doi:10.1093/bioinformatics/btn453.
14. Camin JH, Sokal RR. A Method for Deducing Branching Sequences in Phylogeny. Evolution. 1965. doi:10.2307/2406441.
15. Felsenstein J. Evolutionary trees from gene frequencies and quantitative characters: finding maximum likelihood estimates. Evolution. 1981. doi:10.1111/j.1558-5646.1981.tb04991.x.
16. Felsenstein J. Confidence Limits on Phylogenies: An Approach Using the Bootstrap. Evolution. 1985. doi:10.2307/2408678.

17. Swofford DL. PAUP*. Phylogenetic analysis using parsimony (*and other methods) version 4.0a. Sinauer Associates Sunderland, Massachusetts; 2002.

18. Schliep KP. phangorn: phylogenetic analysis in R. Bioinformatics. 2011. doi:10.1093/bioinformatics/btq706.

19. R Core Team. R: a language and environment for statistical computing: R foundation for statistical computing. 2018. https://r-project.org/.

20. Byun Y, Han K. PseudoViewer: web application and web service for visualizing RNA pseudoknots and secondary structures. Nucleic Acids Research. 2006. doi:10.1093/nar/gkl210.

21. Adl SM, Bass D, Lane CE, Lukeš J, Schoch CL, Smirnov A, et al. Revisions to the Classification, Nomenclature, and Diversity of Eukaryotes. The Journal of Eukaryotic Microbiology. 2019. doi:10.1111/jeu.12691.

Supplementary figures

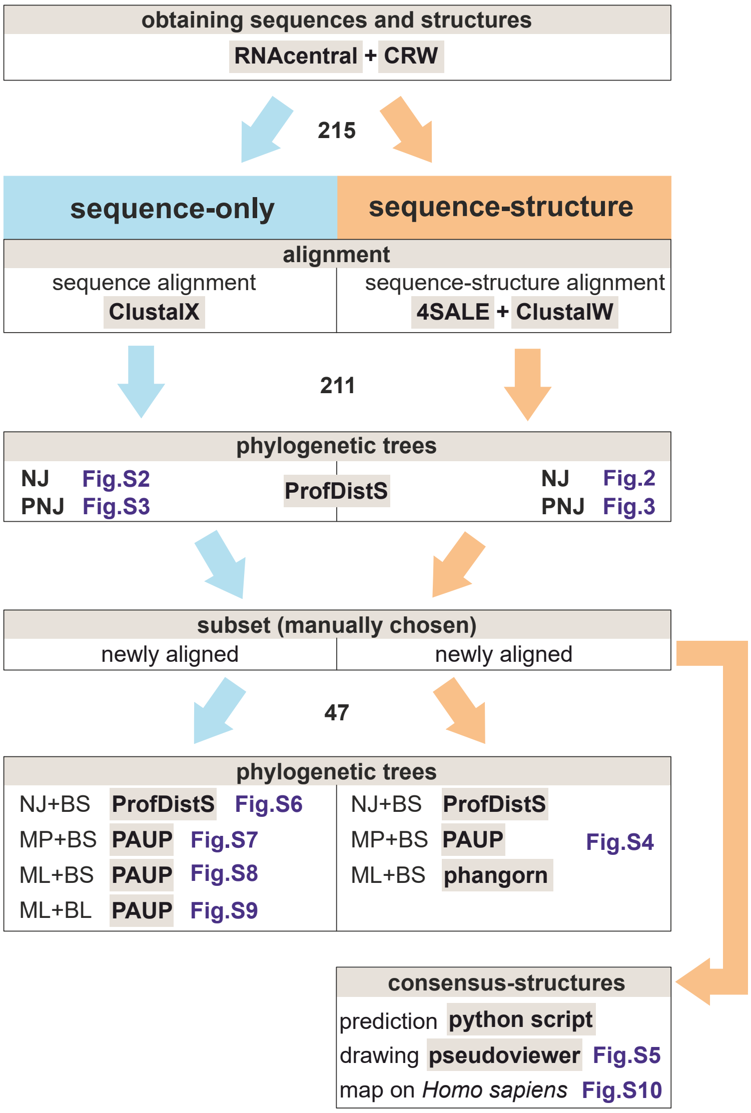

Figure S1: Flowchart of materials and methods. All eukaryote cytosolic 18S ribosomal deoxyribonucleic acid (rDNA) sequences and their individual secondary structures curated by the Comparative RNA Web (CRW) [5] were obtained from RNAcentral [6]. Sequence-only data were aligned with ClustalX [7], whereas sequence-structure data were simultaneously aligned with 4SALE [8, 9]. Four sequences and their structures were removed from the dataset either because of possible contamination or because of uneven length of the sequence and its respective structure. Overall neighbor-joining [10] (NJ) and profile neighbor-joining [11] (PNJ) trees were reconstructed using ProfDistS [12, 13]. A manually chosen subset was further processed through NJ, maximum parsimony [14] (MP) and maximum likelihood [15] (ML) analyses, providing bootstrap [16] (BS) values or branch lengths (BL), using sequence-only as well as sequence-structure data. Using PAUP* [17], ProfDistS and phangorn [18] as implemented in R [19], respective phylogenetic trees were reconstructed. The R script is available at the 4SALE homepage [8]. Using a python script, consensus structures (75 and 100%) were predicted. The 75% consensus structure was drawn using Pseudoviewer [20]. The 100% consensus structure was marked within the 75% consensus figure. Additionally, the consensus structures were mapped on the structure of Homo sapiens, available on RNAcentral [6]. Figure numbers concerning consensus structures and trees are indicated in the flowchart.

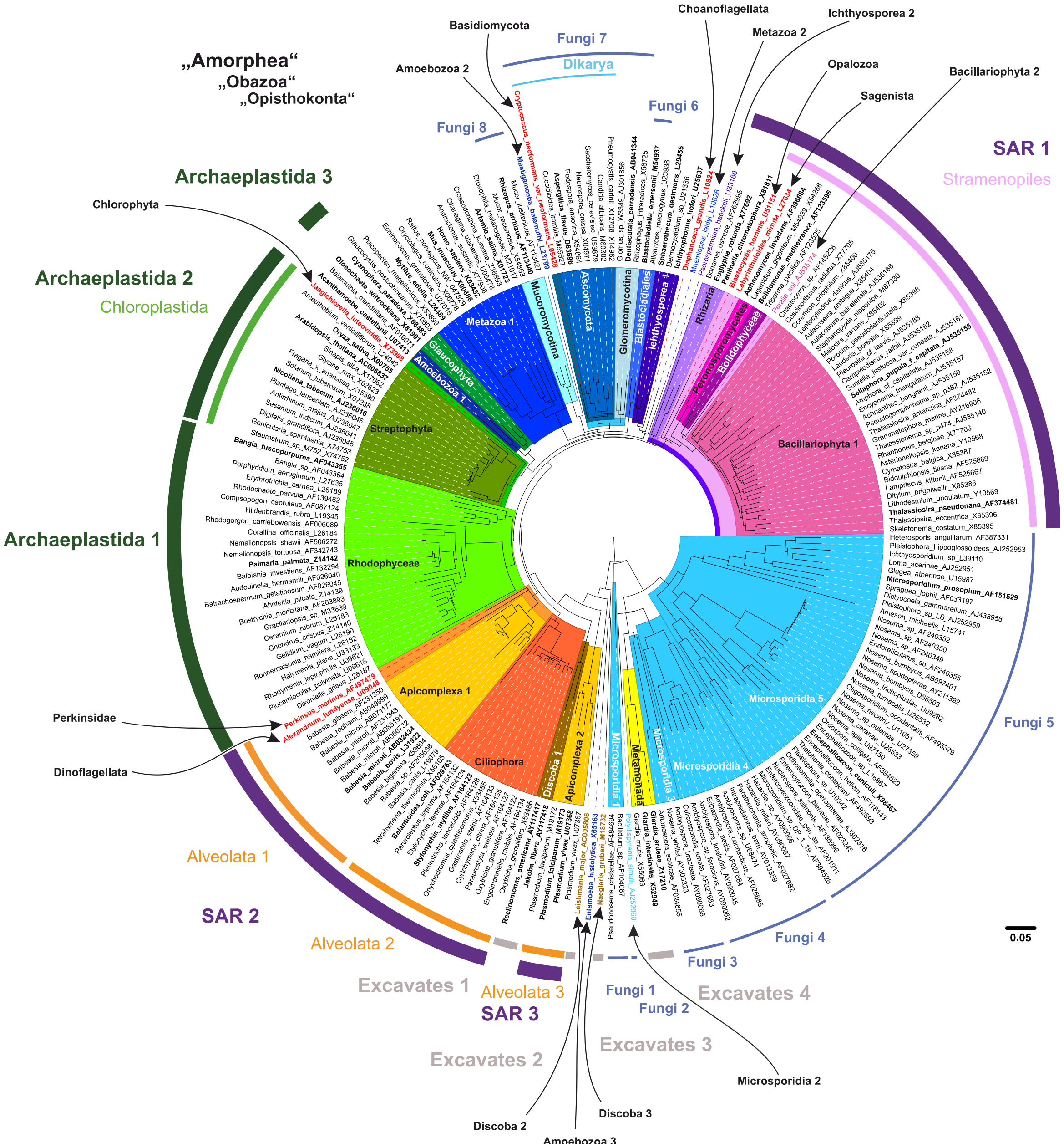

Figure S2: Overall sequence-only neighbor-joining (NJ) tree using the 18S ribosomal deoxyribonucleic acid (rDNA) of all 211 taxa. ClustalX [7] was used for the global multiple sequence alignment. The tree was reconstructed using ProfDistS [12, 13] and midpoint rooted. The scale bar shows evolutionary distances. Taxa names are accompanied by their corresponding GenBank accession number. Clades and respective singular taxa are marked in a color-scheme based on the eukaryotic tree of life published by Keeling and Burki [1]. If clades and singular taxa do not form one monophyletic group, they are numbered consecutively. If a group is only represented by one taxon, the taxon is marked in red. Taxa which were manually selected for the subsampling (Fig. S3 and Figs. S6-S9) are marked bold. Supergroups are indicated according to Burki et al. [2] and Keeling and Burki [1], the names of the supergroups are adapted based on Adl. et al. [21]. With regards to readability the supergroups Amorphea, Obazoa and Opisthokonta are only named once near the majority of the subgroups. The three supergroups are marked with quotation marks since they are not monophyletic. The supergroup Opisthokonta includes Fungi, Metazoa, Choanoflagellata and Ichthyosporea. Amoebozoa are classified as Obazoa.

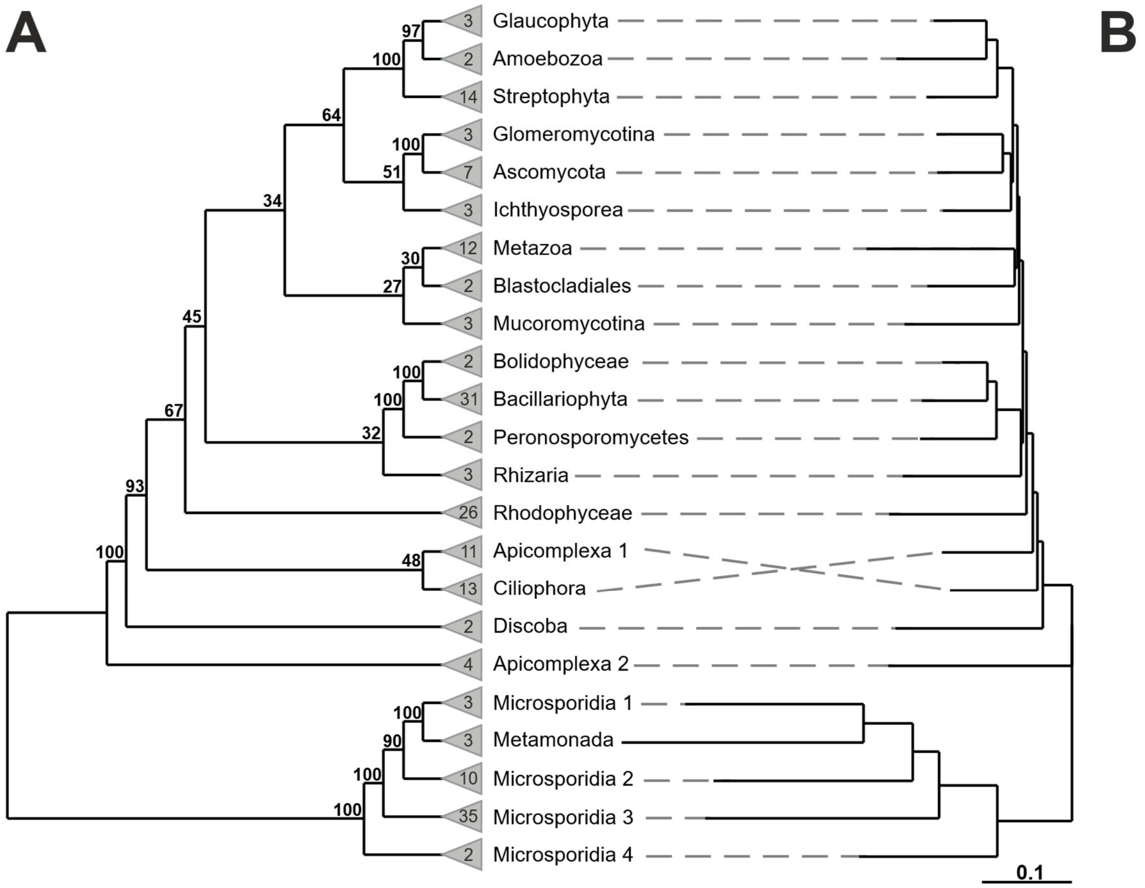

Figure S3: Two-times iterated sequence-only profile neighbor-joining (PNJ) tree with bootstrap (BS) values (A) and original branch lengths (BL) (B). Profiles were predefined according to Fig. S2, singletons were not included. The scale bar shows evolutionary distances. The trees were reconstructed using ProfDistS [12, 13] and rooted according to the overall sequence-only tree (Fig. S2). In each iteration, super-profiles of profiles have been built based on BS values (>75). At internodes, the BS values from 100 pseudo-replicates have been mapped. The numbers in the triangles in front of the taxa represent the quantity of taxa included in the profile.

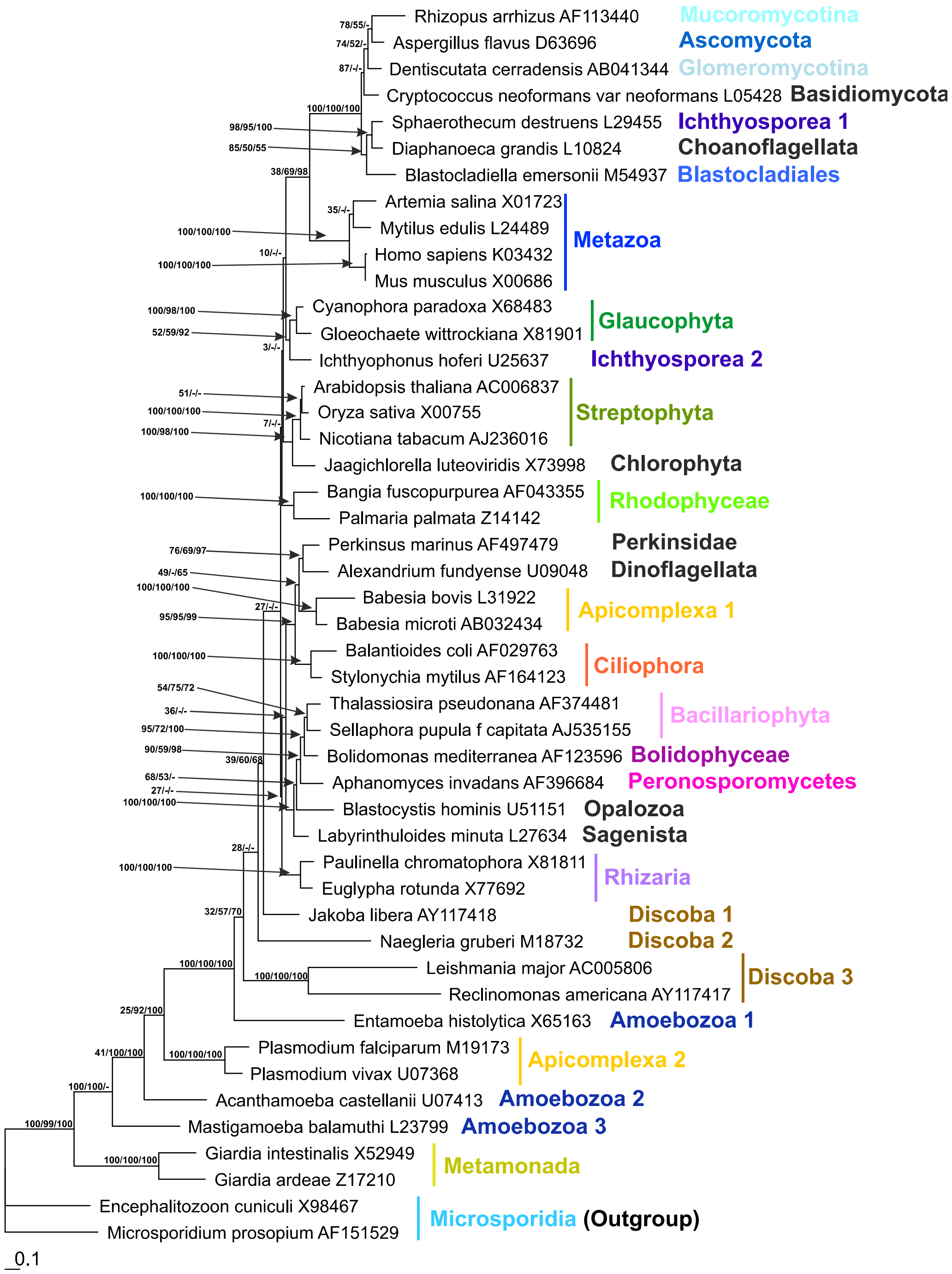

Figure S4: Sequence-structure maximum likelihood (ML) tree with BL using the 18S rDNA of the manually chosen subset of 47 taxa (cf. Figs. 2 and S2). The scale bar shows evolutionary distances. The ML tree was reconstructed using an R script available at the 4SALE homepage [8]. The tree was rooted according to the overall NJ tree (cf. Fig. 2) using *Encephalitozoon cuniculi* and *Microsporidium prosopium* as outgroup. At internodes, the BS values from 100 pseudo-replicates from ML, MP and NJ analyses have been mapped. Varying tree topologies are indicated with "-". The MP tree was reconstructed using PAUP* [17]. The NJ tree was reconstructed using ProfDistS [12, 13]. Taxa names are accompanied by their corresponding GenBank accession number. Monophyletic clades are named alongside the tree according to Burki et al. [2] and Keeling and Burki [1], the names of the supergroups are adapted based on Adl. et al. [21]. In addition they are marked in the same color-scheme like Figs. 2 and S2 which is based on the eukaryotic tree of life published by Keeling and Burki [1]. Polyphyletic clades are numbered consecutively.

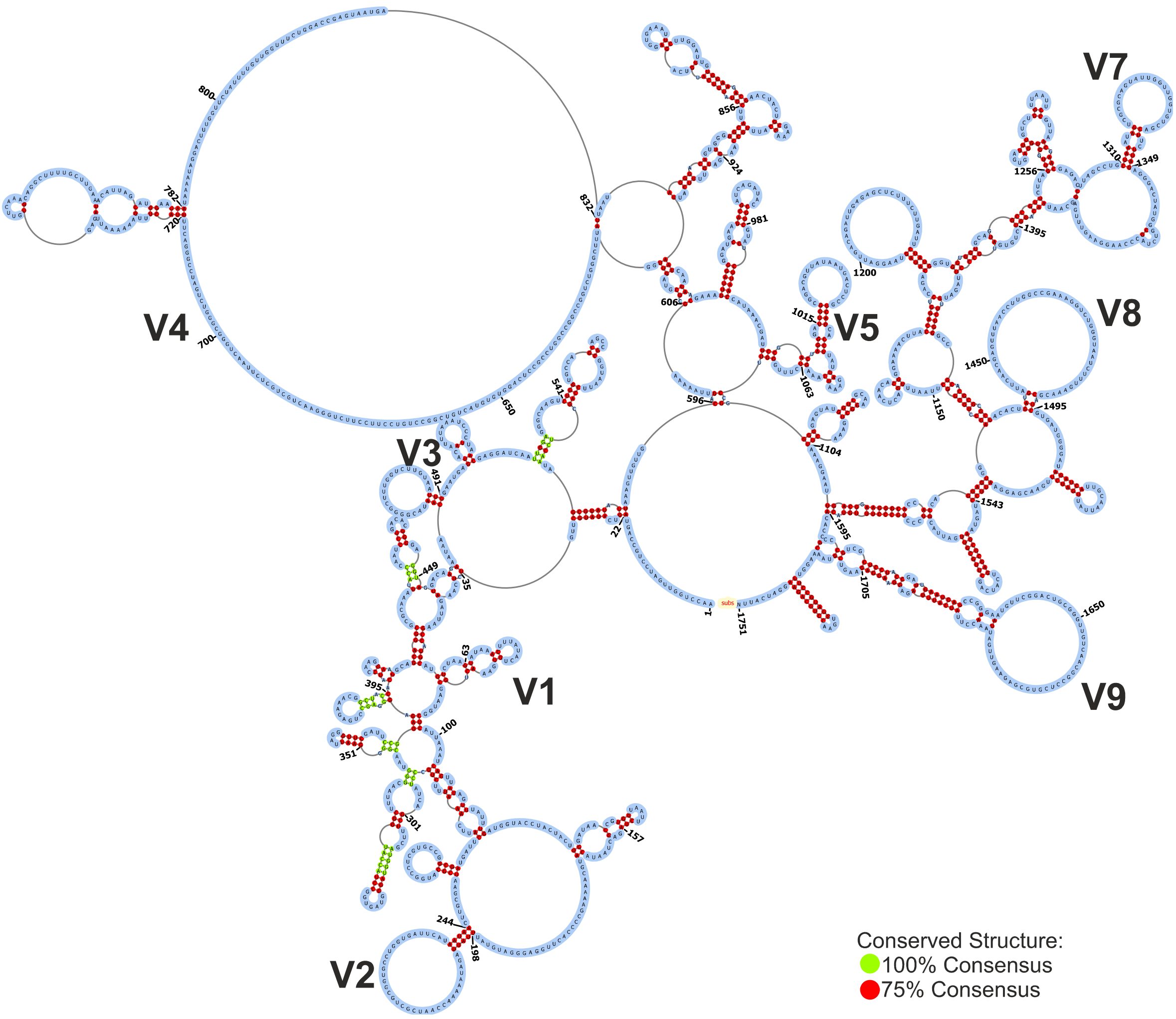

Figure S5: Consensus structures (75 and 100%) of all 47 manually selected organisms. The consensus structures were reconstructed using a python script and based on the sequence-structure alignment as obtained by 4SALE [8, 9]. The 75% consensus structure was drawn using Pseudoviewer [20]. 75% conserved structure is marked with red circles. 100% conserved structure is marked with green circles. Unpaired bases are marked in blue. The helices are numbered according to Dams et al. [3]. The nucleotides are numbered approximately at intervals of 50 in black.

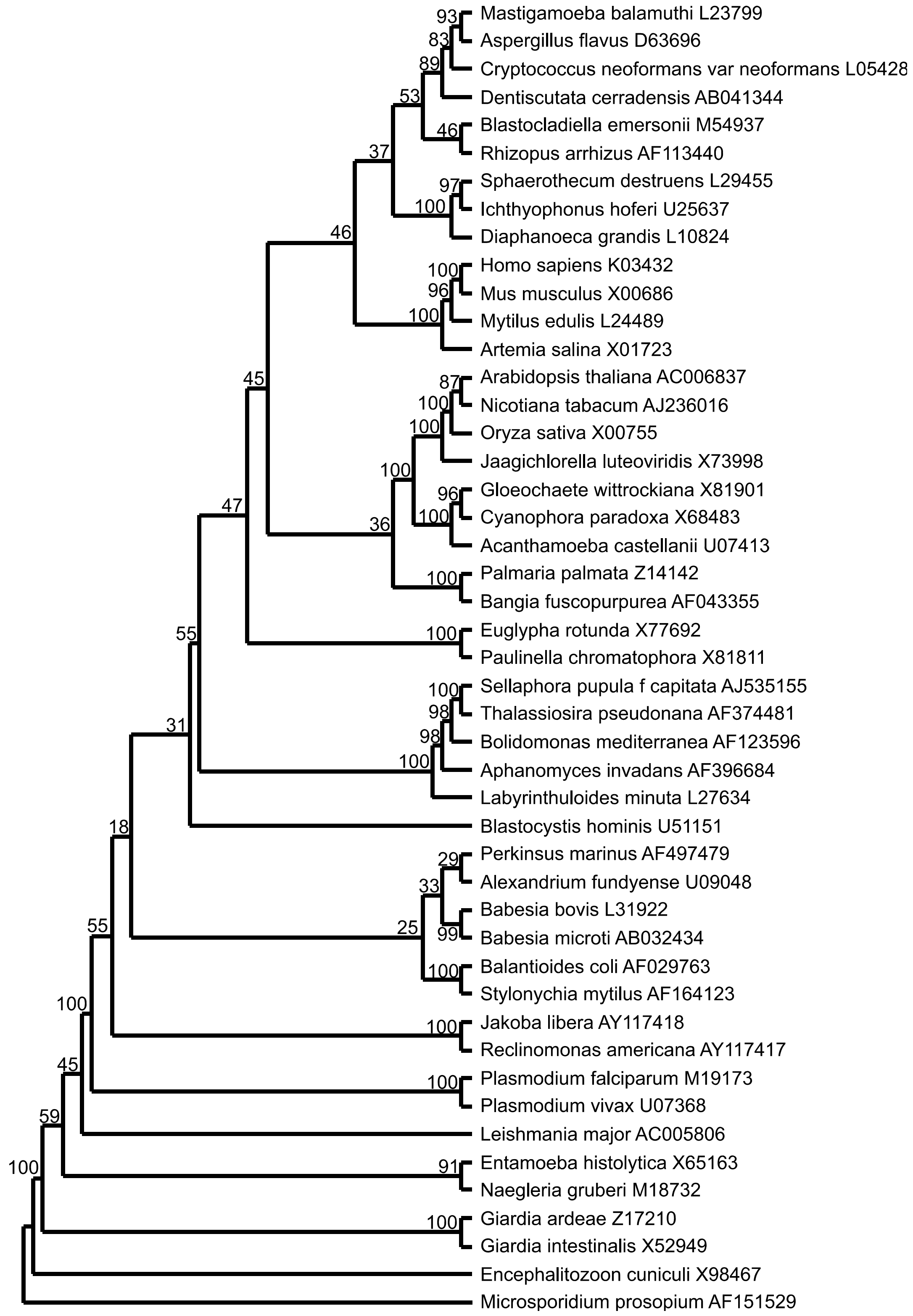

Figure S6: Sequence-only neighbor-joining (NJ) consensus tree using the 18S rDNA of the manually chosen subset of 47 taxa (cf. Figs. 2 and S2). The NJ tree was reconstructed using ProfDistS [12, 13]. The tree was rooted according to the overall tree (cf. Fig. S2) using *Encephalitozoon cuniculi* and *Microsporidium prosopium* as outgroup. At internodes, the bootstrap (BS) values from 100 pseudo-replicates have been mapped. Taxa names are accompanied by their corresponding GenBank accession number.

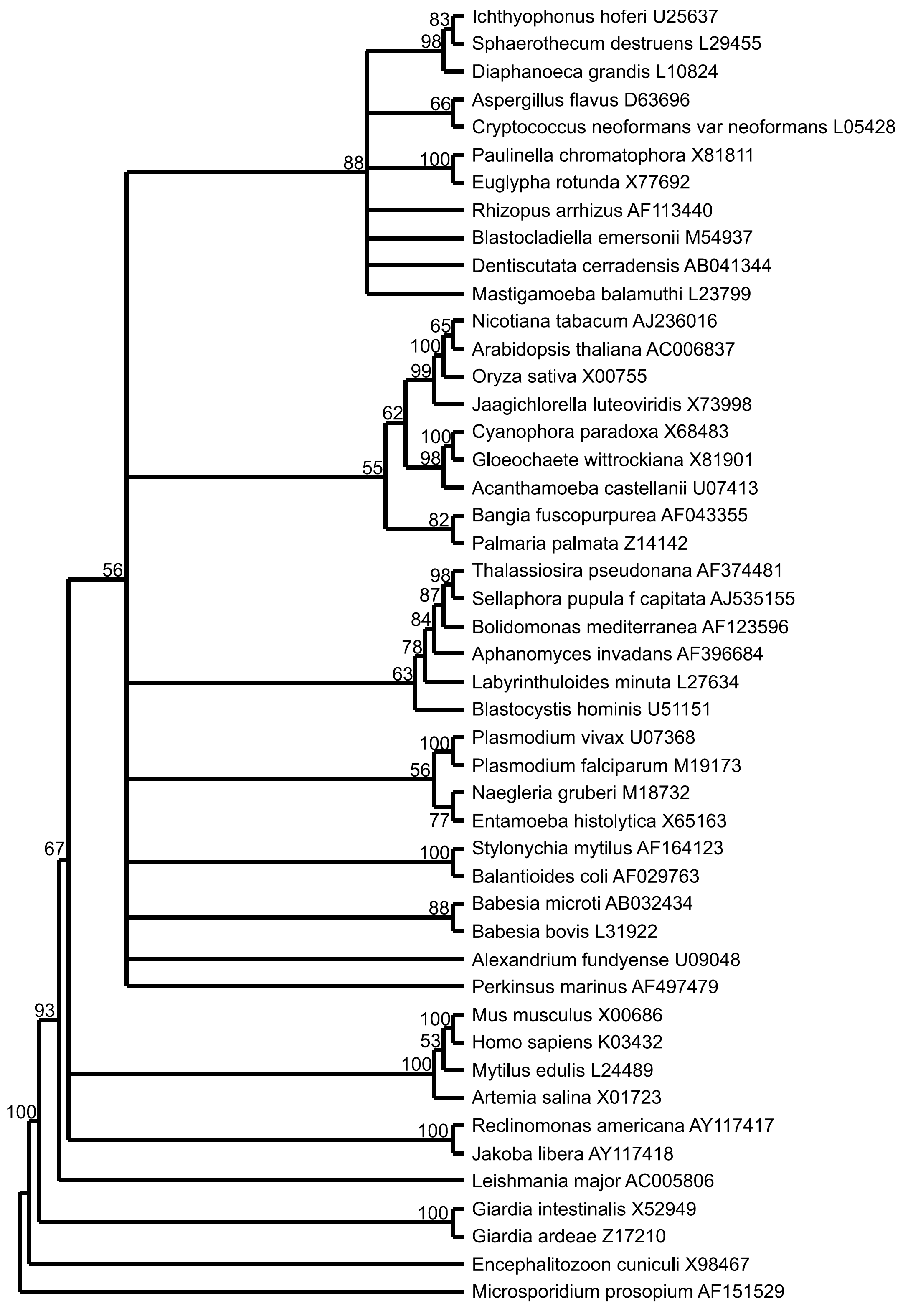

Figure S7: Sequence-only maximum parsimony (MP) tree with BS values using the 18S rDNA of the manually chosen subset of 47 taxa (cf. Figs. 2 and S2). The MP tree was reconstructed using PAUP* [17]. The tree was rooted according to the overall tree (cf. Fig. S2) using *Encephalitozoon cuniculi* and *Microsporidium prosopium* as outgroup. At internodes, the BS values from 100 pseudo-replicates have been mapped. Taxa names are accompanied by their corresponding GenBank accession number.

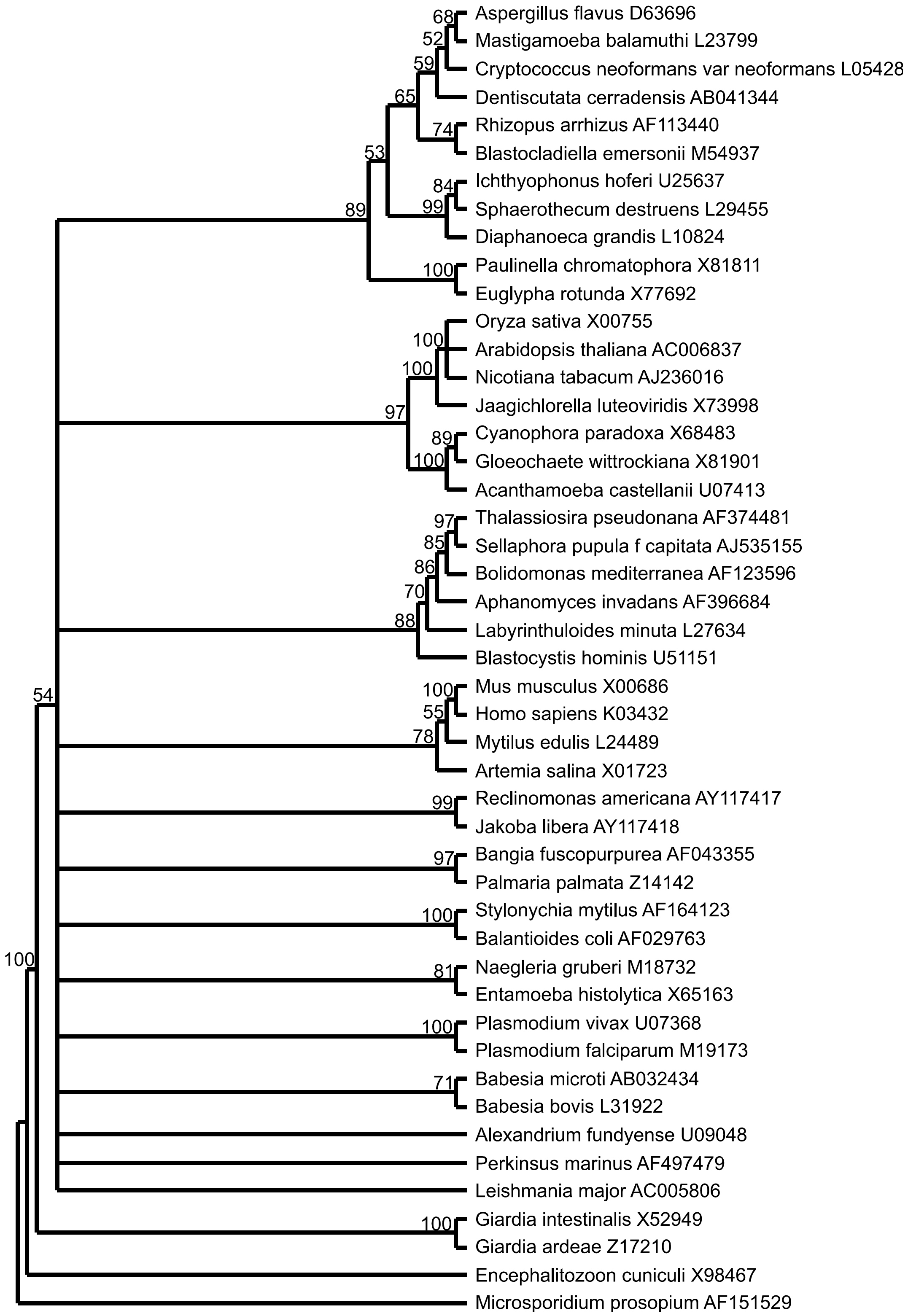

Figure S8: Sequence-only maximum likelihood (ML) tree with BS values using the 18S rDNA of the manually chosen subset of 47 taxa (cf. Figs. 2 and S2). The ML tree was reconstructed using PAUP* [17]. The tree was rooted according to the overall tree (cf. Fig. S2) using *Encephalitozoon cuniculi* and *Microsporidium prosopium* as outgroup. At internodes, the BS values from 100 pseudo-replicates have been mapped. Taxa names are accompanied by their corresponding GenBank accession number.

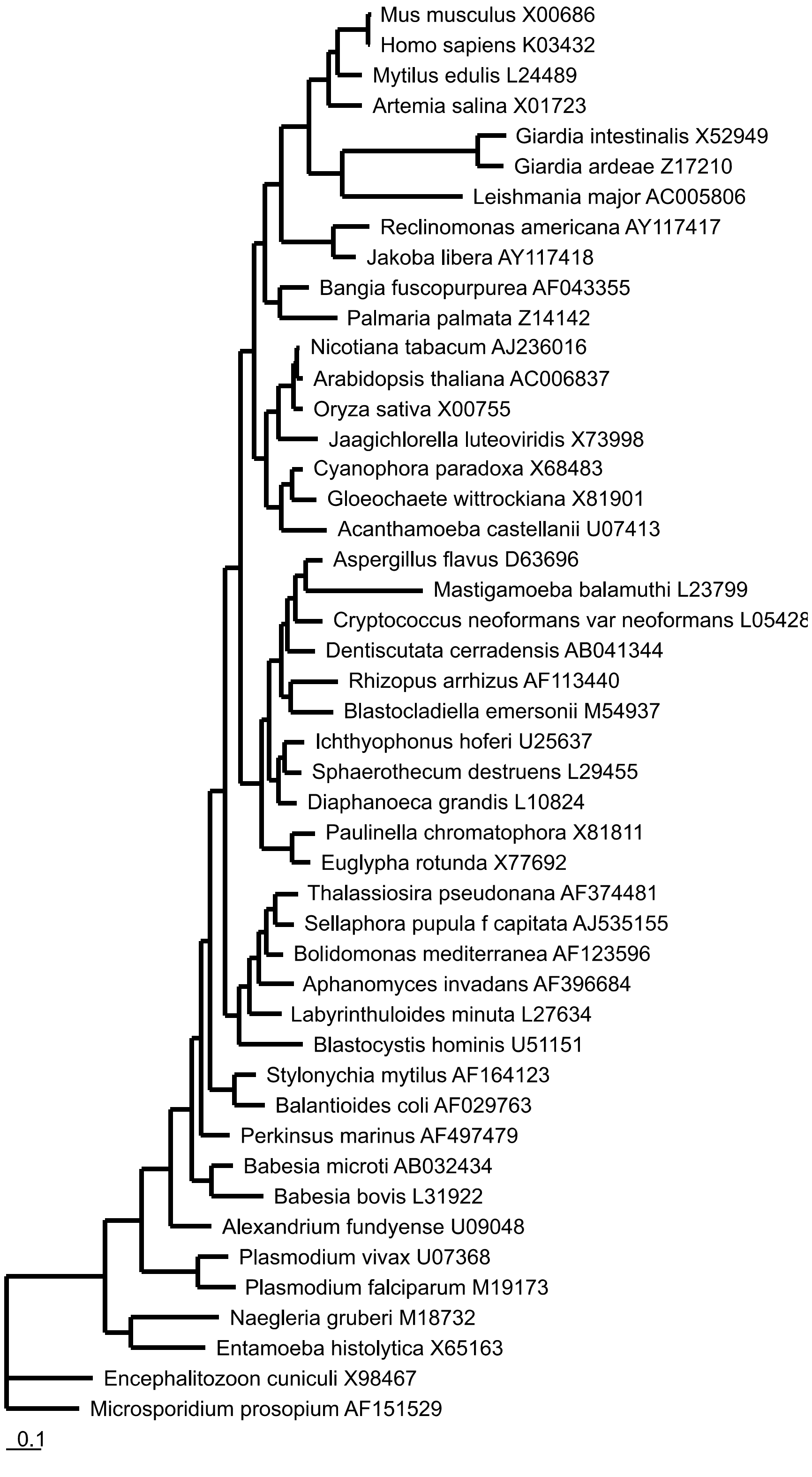

Figure S9: Sequence-only ML tree with branch lengths (BL) using the 18S rDNA of the manually chosen subset of 47 taxa (cf. Figs. 2 and S2). The ML tree was reconstructed using PAUP* [17] The tree was rooted according to the overall tree (cf. Fig. S2) using *Encephalitozoon cuniculi* and *Microsporidium prosopium* as outgroup. The scale bar shows evolutionary distances. Taxa names are accompanied by their corresponding GenBank accession number.

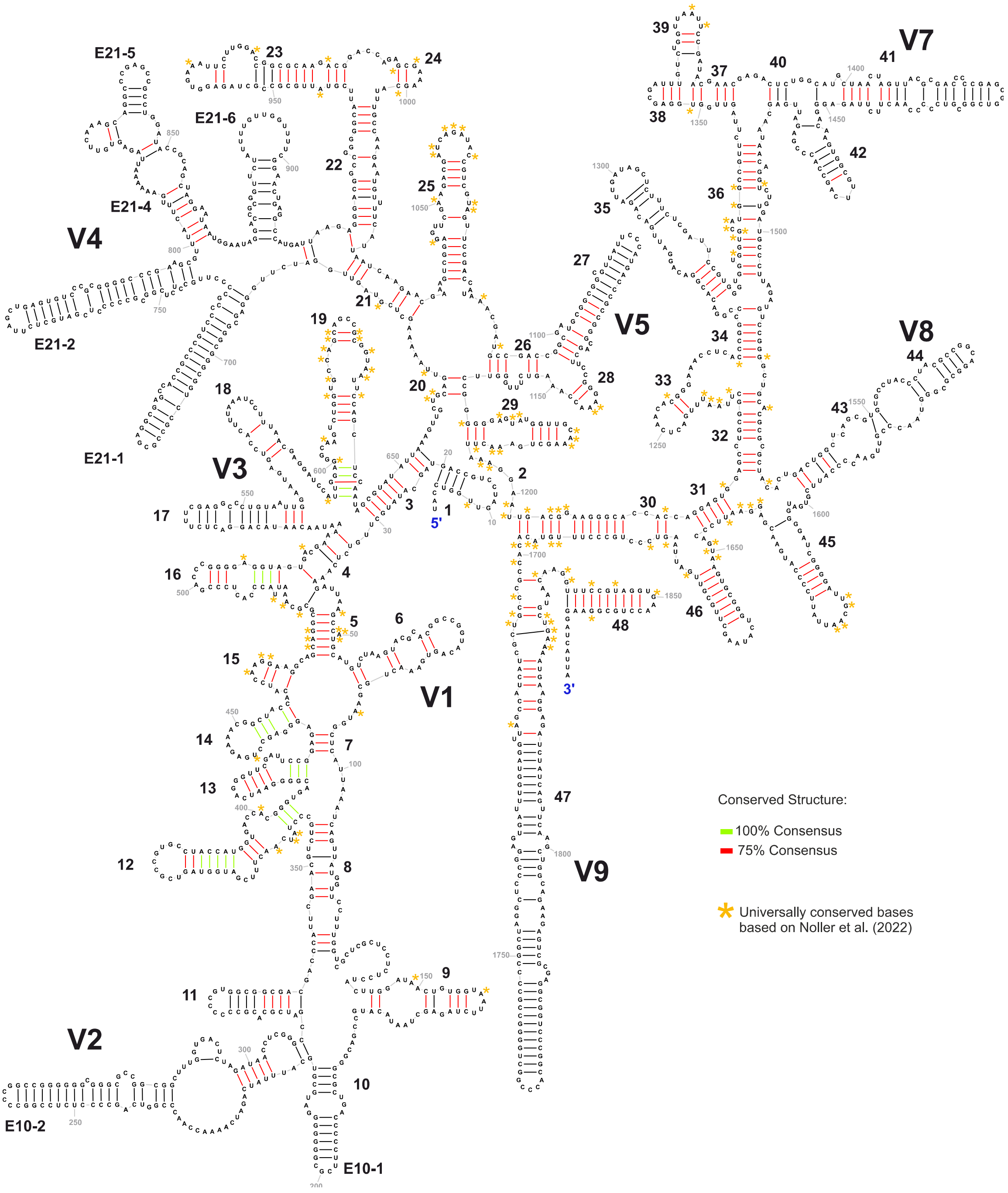

Figure S10: Consensus structures (75 and 100%) of all 47 manually selected organisms mapped on the 18S rDNA secondary structure of Homo sapiens available on RNAcentral [6]. The consensus structures were reconstructed using a python script and based on the sequence-structure alignment as obtained by 4SALE [8, 9]. Conserved structure with 100% consensus is marked in green and conserved structure with 75% consensus is marked in red. In addition, the universally conserved bases according to Noller et al. [4] are indicated with "*". The helices are numbered according to Dams et al. [3]. Nucleotides are numbered in grey in intervals of 50.

Table S1: A list of all used 18S sequences with species names according to RNAcentral and GenBank accession numbers. In total, sequence-structure data for 215 taxa was acquired. Bold taxa and GenBank accession numbers indicate the 47 taxa which were used for the subset analyses. The four taxa, which were removed from the dataset due to uneven length of sequence and the respective structure or possible contamination, are marked in red.

Name of organism	GenBank accession number
**Acanthamoeba castellanii**	**U07413**
Achnanthes bongranii	AJ535150
Ahnfeltia plicata	Z14139
**Alexandrium fundyense**	**U09048**
Allomyces macrogynus	U23936
Amblyospora bracteata	AY090068
Amblyospora connecticus	AF025685
Amblyospora khaliulini	AY090045
Amblyospora sp.	U68474
Amblyospora sp. ferocious	AY090062
Ameson michaelis	L15741
Amphora cf capitellata	AJ535158
Androctonus australis	X77908
Antirrhinum majus	AJ236047
Antonospora scoticae	AF024655
**Aphanomyces invadans**	**AF396684**
**Arabidopsis thaliana**	**AC006837**
Arceuthobium verticilliflorum	L24042
**Artemia salina**	**X01723**
**Aspergillus flavus**	**D63696**
Asterionellopsis kariana	Y10568
Audouinella hermannii	AF026040
Aulacoseira ambigua	X85404
Aulacoseira baicalensis	AJ535186
Babesia bigemina	X59604
**Babesia bovis**	**L31922**
Babesia canis	L19079
Babesia gibsoni	AF231350
Babesia microti	AF231348

**Babesia microti**	**AB071177**
Babesia microti	AB085191
Babesia microti	AB050732
Babesia microti	AB032434
Babesia rodhaini	AB049999
Babesia sp.	AF205636
Bacillidium sp.	AF104087
Balamuthia mandrillaris	AF019071
**Balantioides coli**	**AF029763**
Balbiania investiens	AF132294
**Bangia fuscopurpurea**	**AF043355**
Bangia sp.	AF043364
Batrachospermum gelatinosum	AF026045
Biddulphiopsis titiana	AF525669
**Blastocladiella emersonii**	**M54937**
**Blastocystis hominis**	**U51151**
**Bolidomonas mediterranea**	**AF123596**
Bonamia ostreae	AF262995
Bonnemaisonia hamifera	L26182
Bostrychia moritziana	AF203893
Campylodiscus ralfsii	AJ535162
Candida albicans	M60302
Ceramium rubrum	L26183
Chaetoceros sp.	AF145226
Chalcides guentheri	X61688
Chalcides lanzai	X61689
Chondrus crispus	Z14140
Coccidioides immitis	M55627
Compsopogon caeruleus	AF087124
Corallina officinalis	L26184
Corethron criophilum	X85400
Coscinodiscus radiatus	X77705
Crossodonthina koreana	Z36893

**Cryptococcus neoformans var neoformans**	**L05428**
Culicosporella lunata	AF027683
**Cyanophora paradoxa**	**X68483**
Cymatosira belgica	X85387
Cyrtohymena citrina	AF164135
**Dentiscutata cerradensis**	**AB041344**
Dermocystidium sp.	U21336
**Diaphanoeca grandis**	**L10824**
Dictyocoela gammarellum	AJ438958
Digitalis grandiflora	AJ236045
Ditylum brightwellii	X85386
Dixoniella grisea	L26187
Drosophila melanogaster	M21017
Echinococcus granulosus	U27015
Edhazardia aedis	AF027684
**Encephalitozoon cuniculi**	**X98467**
Encephalitozoon hellem	AF118143
Encephalitozoon sp.	L16867
Encyonema triangulatum	AJ535157
Endoreticulatus sp.	AF240355
Engelmanniella mobilis	AF164134
**Entamoeba histolytica**	**X65163**
Enterocytozoon bieneusi	AF023245
Enterocytozoonidae gen sp.	AF201911
Erythrotrichia carnea	L26189
**Euglypha rotunda**	**X77692**
Euplotes aediculatus	M14590
Fragaria x ananassa	X15590
Gastrostyla steinii	AF164133
Gelidium vagum	L26190
Genicularia spirotaenia	X74753
**Giardia ardeae**	**Z17210**

**Giardia intestinalis**	**X52949**
Giardia muris	X65063
Glaucocystis nostochinearum	X70803
**Gloeochaete wittrockiana**	**X81901**
Glomus sp. W3349	AJ301856
Glugea atherinae	U15987
Glycine max	X02623
Gracilariopsissp.	M33639
Grammatophora marina	AY216906
Halymenia plana	U33133
Hazardia milleri	AY090067
Hazardia sp.	AY090066
Heterosporis anguillarum	AF387331
Hildenbrandia rubra	L19345
**Homo sapiens**	**K03432**
**Ichthyophonus hoferi**	**U25637**
Ichthyosporidium sp.	L39110
Intrapredatorus barri	AY013359
**Jaagichlorella luteoviridis**	**X73998**
**Jakoba libera**	**AY117418**
**Labyrinthuloides minuta**	**L27634**
Lagenidium giganteum	M54939/X54266
Lampriscus kittonii	AF525667
Lauderia borealis	X85399
**Leishmania major**	**AC005806**
Leptocylindrus danicus	AJ535175
Lithodesmium undulatum	Y10569
Loma acerinae	AJ252951
**Mastigamoeba balamuthi**	**L23799**
Melosira varians	X85402
**Microsporidium prosopium**	**AF151529**
Microsporidium sp. DP 1 19	AF394528
Mnemiopsis leidyi	L10826

Mucor lusitanicus	AF113427
Mucor racemosus	X54863
**Mus musculus**	**X00686**
**Mytilus edulis**	**L24489**
Naegleria fowleri	AF338423
**Naegleria gruberi**	**M18732**
Nemalionopsis shawii	AF506272
Nemalionopsis tortuosa	AF342743
Neurospora crassa	X04971
**Nicotiana tabacum**	**AJ236016**
Nosema apis	U97150
Nosema bombycis	AB097401
Nosema bombycis	D85503
Nosema ceranae	U26533
Nosema furnacalis	U26532
Nosema necatrix	U11051
Nosema sp.	AF240349
Nosema sp.	AF240350
Nosema sp.	AF240352
Nosema sp. oulemae	U27359
Nosema spodopterae	AY211392
Nosema trichoplusiae	U09282
Nosema whitei	AY305323
Nucleospora salmonis	AF185996
Okanagana utahensis	U06478
Oligosporidium occidentalis	AF495379
Onychodromus quadricornutus	X53485
Ordospora colligata	AF394529
Orthosomella operophterae	AJ302316
Oryctolagus cuniculus	X06778
**Oryza sativa**	**X00755**
Oxytricha granulifera	AF164122
Oxytricha granulifera	X53486

**Palmaria palmata**	**Z14142**
Paralia sol	AJ535174
Parathelohania anophelis	AF027682
Paraurostyla weissei	AF164127
Paruroleptus lepisma	AF164132
**Paulinella chromatophora**	**X81811**
**Perkinsus marinus**	**AF497479**
Placopecten magellanicus	X53899
Plantago lanceolata	AJ236046
**Plasmodium falciparum**	**M19172**
Plasmodium falciparum	M19173
**Plasmodium vivax**	**U07368**
Plasmodium vivax	U07367
Pleistophora hippoglossoideos	AJ252953
Pleistophora sp.	U10342
Pleistophora sp. LS	AJ252959
Pleurosira cf laevis	AJ535188
Pleurotricha lanceolata	AF164128
Plocamiocolax pulvinata	U09618
Pneumocystis carinii	X12708/X14982
Podospora anserina	X54864
Polydispyrenia simulii	AJ252960
Porosira pseudodenticulata	X85398
Porphyridium aerugineum	L27635
Pseudogomphonema sp. p382	AJ535152
Pseudonosema cristatellae	AF484694
Psorospermium haeckeli	U33180
Rattus norvegicus	NW 047829
**Reclinomonas americana**	**AY117417**
Rhaphoneis belgicae	X77703
Rhizophagus intraradices	X58725
**Rhizopus arrhizus**	**AF113440**
Rhodochaete parvula	AF139462

Rhodogorgon carriebowensis	AF006089
Rhodymenia leptophylla	U09621
Saccharomyces cerevisiae	U53879
**Sellaphora pupula f capitata**	**AJ535155**
Sesamum indicum	AJ236041
Sinapis alba	X17062
Skeletonema costatum	X85395
Solanum tuberosum	X67238
**Sphaerothecum destruens**	**L29455**
Spraguea lophii	AF033197
Staurastrum sp. M752	X74752
Stephanopyxis nipponica	M87330
Stylonychia lemnae	AF164124
**Stylonychia mytilus**	**AF164123**
Surirella fastuosa var cuneata	AJ535161
Tetrahymena thermophila	X56165
Thalassionema sp. p474	AJ535140
Thalassiosira antarctica	AF374482
Thalassiosira eccentrica	X85396
**Thalassiosira pseudonana**	**AF374481**
Thelohania contejeani	AF492593
Triparma pacifica	AF123595
